# Rubber-Composite-Nanoparticle-Modified Epoxy Powder Coatings with Low Curing Temperature and High Toughness

**DOI:** 10.3390/polym15010195

**Published:** 2022-12-30

**Authors:** Run Zhang, Haosheng Wang, Xiaoze Wang, Jian Guan, Meiqi Li, Yunfa Chen

**Affiliations:** 1 State Key Laboratory of Multiphase Complex Systems, Institute of Process Engineering, Chinese Academy of Sciences, Beijing 100190, China; 2 Center of Materials Science and Optoelectronics Engineering, University of Chinese Academy of Sciences, Beijing 100049, China; 3 Key Laboratory of Science & Technology on Particle Materials, Institute of Process Engineering, Chinese Academy of Sciences, Beijing 100190, China

**Keywords:** epoxy-powder coating, rubber composite nanoparticles, tertiary amine, toughness, curing temperature

## Abstract

In this study, a rubber-composite-nanoparticle-modified epoxy powder composite coating with low curing temperature and high toughness was successfully fabricated. The effects of N,N-dimethylhexadecylamine (DMA) carboxy-terminated nitrile rubber (CNBR) composite nanoparticles on the microstructure, curing behavior, and mechanical properties of epoxy-powder coating were systematically investigated. SEM and TEM analysis revealed a uniform dispersion of DMA-CNBR in the epoxy-powder coating, with average diameter of 100 nm. The curing temperature of the epoxy-composite coatings had reduced almost 19.1% with the addition of 1phr DMA-4CNBR into the coating. Impact strength tests confirmed that DMA-CNBR-modified epoxy-composite coatings showed significant improvements compared with the neat EP coating, which was potentially attributed to the nanoscale dispersion of DMA-CNBR particles in epoxy coatings and their role in triggering microcracks. Other mechanical properties, including adhesion and cupping values, were improved in the same manner. In addition, thermal and surface properties were also studied. The prepared epoxy composite powder coating with the combination of low curing temperature and high toughness broadened the application range of the epoxy coatings.

## 1. Introduction

Epoxy resin is an important thermosetting resin that has been used in the manufacture of coatings [1,2,3], adhesives [1,4], electronic materials [1,5], etc. In recent years, epoxy-powder coatings have been widely used as surface functional protective coatings for metal sheets due to their excellent decorative properties [3], good adhesion strength to substrates [3,6], environmental friendliness [7,8], corrosion resistance [9,10,11], and abrasion resistance [6].

Despite these advantages, the applications of epoxy-powder coatings are limited due to their high curing temperatures and brittleness. Therefore, the preparation of modified epoxy-powder coatings to improve their comprehensive performance has become an urgent problem to be solved. In fact, some studies have introduced inorganic nanoparticles, rubber particles, and thermoplastic resins into epoxy resin to improve its mechanical properties [6,12,13,14,15,16,17]. Meanwhile, other studies report that many alkaline curing accelerators such as tertiary amines and nitrogen heterocyclics are also added to epoxy-powder coatings to prepare low-temperature curing-powder coatings. For the mechanical properties, studies have shown that the addition of SiO_2_ nanoparticles has led to significant improvements in the impact strength, tensile strength, and tensile modulus of epoxy-powder coatings [12,18,19]. Nano-CaCO_3_-modified powder coatings are found to have significantly improved the cupping properties and tensile properties compared with unmodified powder coatings [20,21]. Diego et al. [22] report that the incorporation of MMT particles in the formulation of epoxy-powder coatings provided good adhesion and thermal properties. There have been many studies that have reported significant increases in fracture toughness with the addition of rubber in epoxy composite materials [16,23,24]. However, there have been few reports about rubber-modified epoxy-composite coatings.

The high curing temperature of epoxy-powder coatings limits their application in heat-sensitive substrates. Curing accelerators are a class of substances that can effectively reduce the curing temperature and reaction activation energy, and shorten the curing time [25,26]. Basic compounds such as nitrogen heterocyclics and tertiary amines are able to provide higher reactivity in smaller amounts in epoxy composite materials. 2-MI is widely used in epoxy-powder coating systems due to its relatively high reactivity, relatively low odor, and solid properties at room temperature. However, 2-MI has a high melting point and low compatibility with resin matrix, which limits its application. Tertiary amine is another highly effective curing accelerator for epoxy. The facilitating effect of tertiary amines on the curing reaction is related to the density of electrons on nitrogen atoms in their molecular structure and the length of molecular chains [27,28]. However, the short chain liquid tertiary amine catalysts have high catalytic activity and could cure liquid-phase epoxy resins at room temperature, which are not suitable for solid epoxy-powder coating.

Up to now, there is no study to propose a solution that can solve both the brittleness and high curing temperature of epoxy coatings. In order to overcome the brittleness and high curing temperature of epoxy-powder coatings, this study investigated the effect of DMA-CNBR as a toughening modifier and curing promoter in epoxy-powder coatings. Carboxy-terminated nitrile rubber (CNBR), a kind of powder rubber with an average size of 100 nm, was prepared by the combined technologies of irradiation and spry drying [29], while DMA was a liquid long chain tertiary amine at room temperature. In this work, rubber particles were innovatively adsorbed long-chain liquid tertiary amines, and liquid tertiary amine curing promoters were successfully applied to epoxy-powder coatings. Transmission electron microscopy (TEM) and scanning electron microscopy (SEM) revealed that the DMA-CNBR achieved nanoscale dispersion in epoxy coatings. The comprehensive properties of the epoxy-powder coating with different DMA-CNBR contents were also studied. The mechanical (impact, adhesion strength, and cupping value), thermal properties, and surface properties of epoxy-powder coatings were examined to consider the influence of CNBR-DMA, while the curing behavior of the coatings was studied by differential scanning calorimetry (DSC). In this paper, a new and low-cost epoxy powder nanocomposite coating industrial manufacturing strategy is proposed, which provides important enlightenment for the practical application of nanocomposite powder coating.

## 2. Experimental

### 2.1. Materials

Commercial bisphenol A-based epoxy resins 604 (epoxy value 0.12 mol/100 g) were supplied by the Anhui Hengyuan Group (Anhui, China). The curing agent dicyandiamide was supplied by Sinopharm Chemical Reagent Co., Ltd. (Shanghai, China). A defoaming agent (benzoin) and a flatting agent 588 were provided by South Sea Chemical (Ningbo, China). Carboxy-terminated nitrile rubber (CNBR) nanoparticle was supplied by the Beijing Research Institute of Chemical Industry (Beijing, China). The N,N-dimethylhexadecylamine (DMA) was purchased from Beijing Waokai Technology Co., Ltd (Beijing, China).

### 2.2. Preparation of DMA-CNBR

The composite DMA-CNBR was prepared by mechanically mixing carboxy-terminated nitrile rubbers and liquid tertiary amine. In this process, different amounts of liquid tertiary amine were absorbed onto carboxy-terminated nitrile rubbers. The mass ratio of tertiary amine to rubber was 1:4, abbreviated as DMA-4CNBR.

### 2.3. Preparation of the DMA-CNBR/EP

The epoxy coating modified by DMA-CNBR was prepared as follows. In the first step, the quality of each component of the DMA-CNBR/EP hybrid system was accurately weighed, including epoxy resin, dicyandiamide, DMA-CNBR, additives, etc. The content of each component in the DMA-4CNBR/epoxy nanocomposite coating systems was listed in Table 1. In the second step, the weighed components were added to the high-speed mixer to obtain a homogenously dispersed material, which was transported to the twin-screw mixer (SLJ-30E, Donghui,) through the feeder, so that the various components were mixed under certain temperature conditions. The temperature in the twin screw extruder was set as follows: T_S1_-90 °C, T_S2_-100 °C, T_S3_-110 °C, and T_S4_-120 °C. The blending material was cooled to room temperature and pressed into sheets through a roller machine. Then, this kind of sheet material was pulverized in the whirlwind ultrafine pulverizer (ACM mill-02D, Donghui) with 1500 revolutions per min. The milled powder coating was screened through a screen mesh with a mesh size 160 mesh. The powder coatings were sprayed by an electrostatic spray gun (OptiStar Type CG13) with an output voltage of 60 kV to the tinplate plates. Finally, the sprayed coated samples were cured at 180°C for 10 min and cooled, and their properties were investigated.

### 2.4. Coating Characterization

The cross-section morphologies of epoxy coating samples were determined by SEM (Japan Electronics-Oxford JSM-7800(Prime), Tokyo, Japan) and TEM (Japan JEOL JEM-F200, Tokyo, Japan). Samples should be prepared by freezing sections at −80 °C and stained with OsO_4_ before TEM observation.

In situ diffuse reflectance infrared spectroscopy (in-situ DRIFTs, Bruker Vertex 70) was used to test the characteristic absorption peak of the DMA-CNBR/EP coating at 180 °C. Atomic force microscopy (AFM, AFM Multimode 8 microscope with electronics nanoscope V, Bruker, Saarbrücken, Germany) was used to determine the surface roughness of the epoxy coatings. Root mean square (Rq) indicates surface roughness.

The DSC results can be used to evaluate the thermal curves of epoxy-powder coatings during the curing process. The curing temperature and glass transition temperature (T_g_) of DMA-CNBR/epoxy-powder coating were measured using the DSC (TA Instruments Q1000, Newcastle, DE, USA), where the 3–7 mg powder coating samples were put into aluminum crucible under a nitrogen atmosphere with a flow rate of 20 mL /min and a heating rate of 10 K/min. T_g_ of cured epoxy-powder coating sample was calculated from the second cycle. To measure the T_g_ of the sample, the sample was heated to 250 °C at a heating rate of 10 K/min, cooled to room temperature, and then reheated to 150 °C at a heating rate of 10 K/min in the DSC instrument. The gelation time of the powder coatings was measured by the multi-function gelation time meter (CQ-J20). The impact strength of DMA-CNBR/epoxy-powder coating was measured by an impact meter (Elcometer 1615) according to ASTM D2794. The adhesion strength of the epoxy coatings was measured by an automatic pull-out adhesion tester (Positest AT-A, DeFelsko, New York, NY, USA). The cupping value of the epoxy-powder coatings was determined by a cupping-tester (Pushen 2705).

## 3. Results and Discussion

### 3.1. Characterization of the CNBR and DMA-CNBR/EP

**Figure 1:**
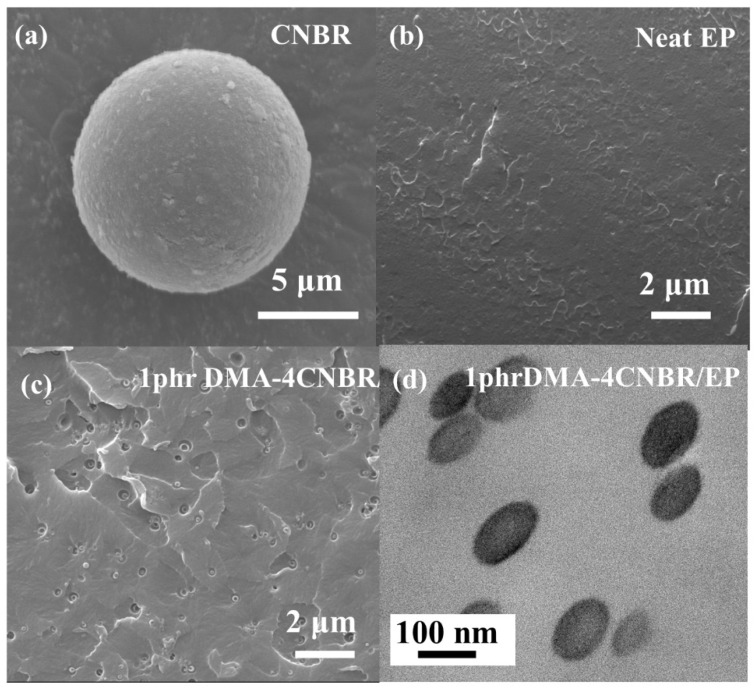
SEM (**a**) morphology of CNBR. SEM (**b**,**c**) and TEM (**d**) morphology of neat epoxy coating and 1phr DMA-4CNBR/EP coating cross-section.

SEM is used to study the microstructure and morphology of CNBR and DMA-CNBR/EP. Figure 1a shows that CNBR existed in the form of large spheres with a diameter of over 10 μm, which was several times larger than that of the primary rubber particle. DMA-CNBR was obtained by the adsorption of liquid tertiary amine with CNBR, as shown in the figure. Under high shear forces, agglomerated particles could be redistributed into individual nanoparticles, and DMA is also dispersed into the matrix. SEM and TEM micrographs of neat-epoxy-coating and 1phr DMA-4CNBR/EP-coating cross sections are shown in Figure 1b–c. The neat epoxy coating had a relatively smooth morphology (Figure 1b). The DMA-4CNBR, with an average diameter of 100 nm, was uniformly dispersed in the epoxy coating without agglomeration (Figure 1c,d). This means that rubber composite nanoparticles could achieve uniform dispersion in the powder-coating melt-mixing process. This is caused by the good compatibility between epoxy and CNBR, and the special crosslinking structure of rubber nanoparticles. This special structure can not only ensure the elasticity of rubber particles but also will not lead to agglomeration between rubber particles, so the nano rubber particles can be dispersed in the epoxy coating in the form of a single particle [15,30].

### 3.2. Curing Behaviors and Curing Kinetics

**Figure 2:**
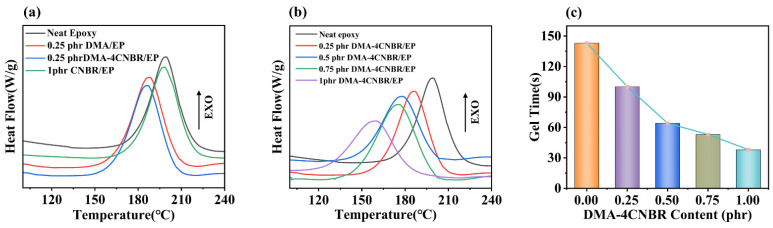
(**a**,**b**) DSC curves for different coatings at 10 °C/min (**c**) Gelling time of different proportions of DMA-4CNBR-modified epoxy coating.

In this work, liquid tertiary amines were adsorbed by rubber particles and applied to epoxy-powder coatings. The performance of different epoxy-coating systems is closely related to their curing temperature and curing behavior [8,23,31,32]. Epoxy coating systems that can be cured at lower temperature result in higher reactivity. The curing behaviors of epoxy powders of different systems were tested by DSC, as shown in Figure 2. After an equal amount of DMA was adsorbed by rubber particles, the curing promotion effect was better than that of pure DMA. Compared to neat epoxy-powder coating, the curing temperature of epoxy powder doped with 1phr CNBR diminished slightly. The curing temperature of 0.25phr DMA/EP and 0.25phr DMA-4CNBR/EP occurred at 188.0 °C and 185.7 °C, respectively. As mentioned above, for the epoxy coating initiated by DMA-4CNBR, the curing temperature could drop even more because the catalytic active of DMA-4CNBR was higher than that of DMA.

The curing temperatures of different contents of DMA-4CNBR-modified epoxy coating were evaluated by DSC analysis under nitrogen atmosphere. According to Figure 2b, with the enhancement of DMA-4CNBR content, the peak curing temperature (T_p_) of DMA-4CNBR/EP systems decreased gradually under the same heating rate, indicating that DMA-4CNBR could enhance the reactivity of DMA-4CNBR/EP systems. The neat epoxy coating had a peak curing temperature at 202.6 °C. The DMA-4CNBR prepared in this paper could significantly reduce the curing reaction temperature of epoxy-coating systems. The 0.25phr DMA-4CNBR/EP and neat epoxy coating occurred at 185.7 and 202.6 °C, respectively, indicating that the curing reaction occurred rapidly with the addition of DMA-4CNBR. When the catalyst DMA-4CNBR addition was increased to 1 phr, the sample curing temperature was further reduced to about 164.1 °C. Figure 2c exhibited a comparison of the gelling time of different proportions of DMA-4CNBR-modified epoxy coating. The gelling time reflects the speed of the epoxy curing reaction. As the DMA-4CNBR/EP ratio increased, the gelling time decreased. It is further demonstrated that DMA-4CNBR could speed up the curing reaction of epoxy coatings and had low temperature curing.

**Figure 3:**
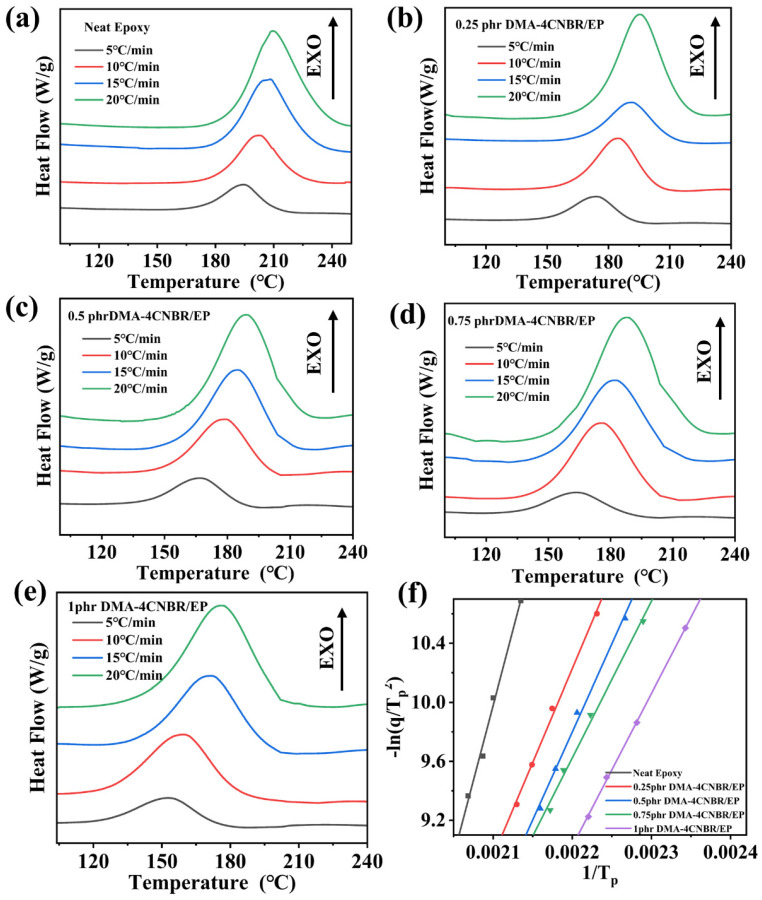
DSC thermograms of (**a**) neat epoxy, (**b**) 0.25phr DMA-4CNBR/EP, (**c**) 0.5phr DMA-4CNBR/EP, (**d**) 0.75phr DMA-4CNBR/EP, and (**e**) 1phr DMA-4CNBR/EP, at different heating rates. (**f**) The correlation fitting curves of different content DMA-4CNBR modified epoxy resin systems based on Kissinger’s theories.

The DSC was used to measure the non-isothermal curing behaviors of different systems (neat epoxy coating and different contents of DMA-4CNBR-modified EP) under different heating rates. Figure 3 clearly showed that with the enhancement of DMA-4CNBR content, the T_p_ of DMA-4CNBR/EP systems gradually decreased at the same heating rate, indicating that DMA-4CNBR could accelerate the curing reaction of the coating. The activation energy (E_a_) can react to the activity of the curing reaction. Kissinger’s theories can be used to calculate the neat epoxy coating and different contents of DMA-4CNBR modified EP systems [32,33]. The theories can be expressed by the following Equation (1):

(1) $$-\ln\left(\frac{q}{T_{p}^{2}}\right)=\frac{\mathrm{Ea}}{{\mathrm{RT}}_{p}}-\ln\left(\frac{\mathrm{AR}}{E_{a}}\right)$$

where q is the heating rate (°C /min), T_p_ is the peak temperature during the curing process (K), R is the gas constant, and A is the pre-exponential factor (min^−1^). The E_a_ could be gained from the slope of the line obtained by linear fitting based on Kissinger’s method shown in Figure 3f. Table 2 listed the E_a_ values calculated by Kissinger’s method. Apparently, the activation energy of 1phr DMA-4CNBR/EP is 86.1 kJ/mol, nearly half of neat epoxy coating (165.4 kJ/mol), indicating the superior catalytic performance of DMA-CNBRs. The carboxyl and nitrile group in CNBRs and the long-chain tertiary aminas could catalyze the epoxy curing reaction, resulting in a decrease in the apparent activation energy of the system [8].

### 3.3. Mechanical and Thermal Performance

**Figure 4:**
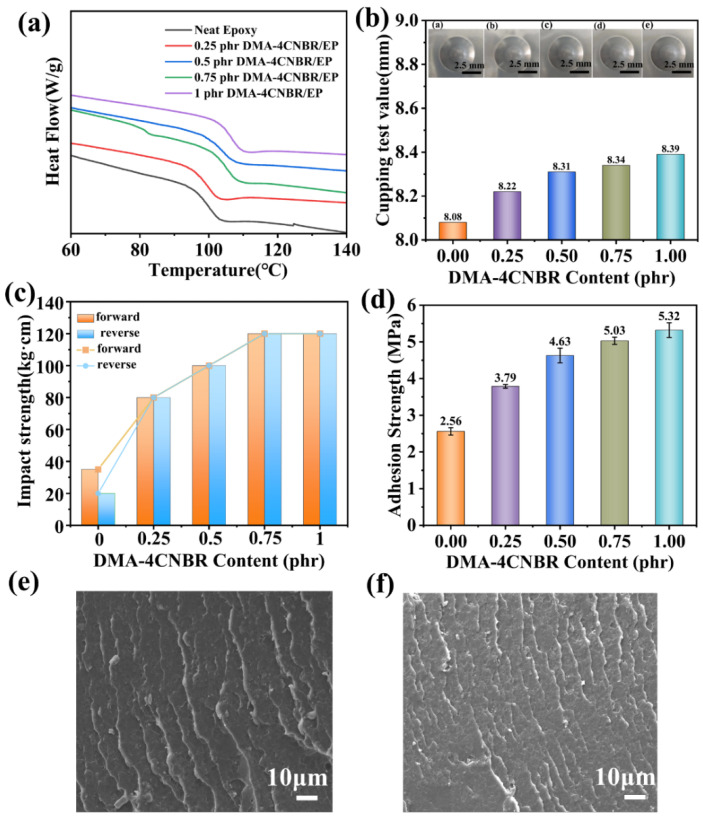
(**a**) DSC curves of the neat epoxy and the DMA-CNBR-modified composite epoxy coating after second heating; (**b**) cupping results of the epoxy-powder coatings; (**c**) impact strength of the epoxy resin coatings loaded with different contents of DMA-4CNBR; (**d**) adhesion test results of the nanocomposite coating with different addition amounts of DMA-4CNBR; (**e**,**f**) SEM photographs of fracture surface of neat epoxy coating and 1phr DMA-4CNBR-modified epoxy coating.

Impact strength is an important indicator to evaluate the mechanical properties of the coating, which is used to evaluate the impact strength of the coating or to judge the brittleness and toughness of the coating. An impact machine was used to measure the impact strength of different contents of DMA-4CNBR-modified epoxy coatings. The experimental results were shown in Figure 4c, and the toughness of the epoxy coating modified by 0.75phr DMA-4CNBR was dramatically increased by 242% from 35 kg·cm to 120 kg·cm, compared with the neat epoxy coating. The improvement of the impact strength was due to several factors, including the uniform dispersion of DMA-CNBR in epoxy coatings, the number of DMA-CNBR in epoxy coatings, and the theory of microcracks. When the coating was impacted by external forces, the DMA-CNBR could play a role in triggering microcracks to absorb the impact energy, thereby improving the impact resistance of the coating. SEM photographs of the impact fracture surface of the neat epoxy coating and the DMA-4CNBR-modified epoxy coating are shown in Figure 4e–f. As shown in Figure 4e, the average distance between microcracks is over 5 μm on the cross-section of neat epoxy coating. However, it can be observed that the average distance between microcracks was only about 2 μm on the cross-section of DMA-4CNBR/EP, as shown in Figure 4f. Therefore, due to the smaller distance between microcracks, there were more microcracks on the cross-section of DMA-4CNBR/EP than on the neat epoxy coating. As we all know, microcracks are the main toughening mechanism of thermosetting resins [34]. Therefore, it was explanatory that the DMA-4CNBR toughened the epoxy coating where rubber particle size was about 100 nm, had many more microcracks, and had a much higher impact strength than the neat epoxy coating.

The adhesion test is the basic indicator for detecting the good mechanical properties of the coating and the metal interface. Good adhesion can play a role in blocking the corrosive medium from invading the matrix, avoiding the peeling of the coating caused by the long-term erosion of the corrosive medium. Adhesion drawing experiments were used to evaluate the adhesion strength between the prepared coating and the metal matrix. The adhesion test results of the composite coating with different amounts of DMA-4CNBR added are shown in Figure 4d. As can be seen from the Figure 4d, with the increasing content of DMA-4CNBR, the adhesion of the DMA-4CNBR/epoxy composite had an exaltation compared with the neat epoxy coating, whose adhesion strength was 2.6 MPa. For 1phr DMA-4CNBR/EP, the adhesion strength increased to 5.3 MPa and was strengthened by 107.8%. The improvement of the adhesion strength was mainly due to the strong reactivity of DMA-CNBR and the nanoparticles’ reaction with the active group of epoxy resin, which increased the bond strength of the coating with the aluminum substrate, thereby improving the adhesion of the coating. The adhesion results showed that the addition of DMA-4CNBR could enhance the binding strength of the coating and the metal matrix, and the strength of the binding was related to the addition of DMA-4CNBR.

The cupping test is mainly used to evaluate the resistance of the epoxy coating to dry-cracking or separation from metal substrates after it is gradually deformed under standard conditions. It is generally believed that the higher the cupping value, the better the forming performance of the epoxy coating. The results of the cupping experiment are shown in Figure 4b, where the coating samples form convex hulls in the central area and cracks appeared in their walls. As the DMA-CNBR content increased, the cupping value increased. The results show that the 1phr DMA-4CNBR/EP coating had good toughness at room temperature and did not have brittle fracture when deformed.

DSC is used to study the thermal behavior of prepared composite coatings and to study the effect of DMA-CNBR on the heat resistance of epoxy-powder coatings. Figure 4a showed the DSC curve of a composite coating with different amounts of DMA-CNBR after secondary heating. The T_g_ of 1phr DMA-4CNBR/EP was raised by 4.7% from 95.4 °C to 100.0 °C, compared with the neat EP coating. The results demonstrated that DMA-4CNBR could improve the heat resistance of epoxy systems. As reported in Huang F, despite the fact CNBR was introduced in the anhydride -cured epoxy system, its heat resistance showed, instead of a decrease, an increase of 3.5% from 136.1 °C to 140.9°C, in terms of T_g_ [4,35]. The role of DMA-CNBR in epoxy/amine systems has been rarely reported. In this work, when toughened with DMA-CNBR, the T_g_ of the epoxy/amine system not only did not decrease but increased by 4.5 °C.

### 3.4. The Surfaces of Epoxy-Coating Systems Analysis

**Figure 5:**
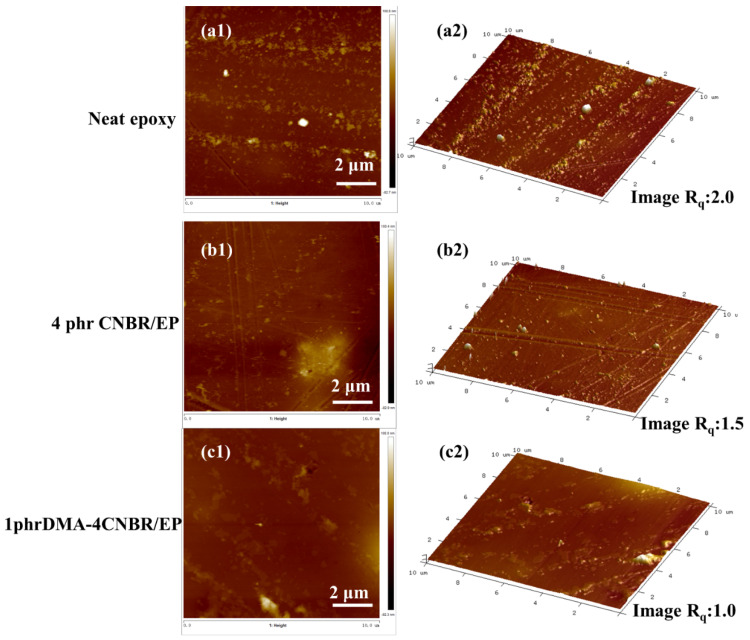
AFM micrographs of the coatings: (**a1**–**c1**) the 2D topographical characteristics of AFM; (**a2**–**c2**) the 3D topographical characteristics of AFM.

The surfaces of coating samples prepared from different epoxy powder systems were examined by AFM, and the resulting images are shown in Figure 5. The 2D and 3D topographical characteristics of the neat epoxy coatings and other epoxy powders systems coatings are shown in Figure 5. The average roughness of the test area is expressed in R_q_ [6]. As shown in Figure 5, the R_q_ value of neat epoxy was 2.0 nm when 1phr DMA-4CNBR/epoxy-modified epoxy coating, and the R_q_ value of the coating was the lowest, which was 1.0 nm. The reduction in the R_q_ value by the addition of the 1phr DMA-4CNBR into the epoxy coating indicated that the rubber composite nanoparticles made the epoxy-composite coating superior to the neat epoxy coating.

### 3.5. Curing Promoting Mechanism

**Figure 6:**
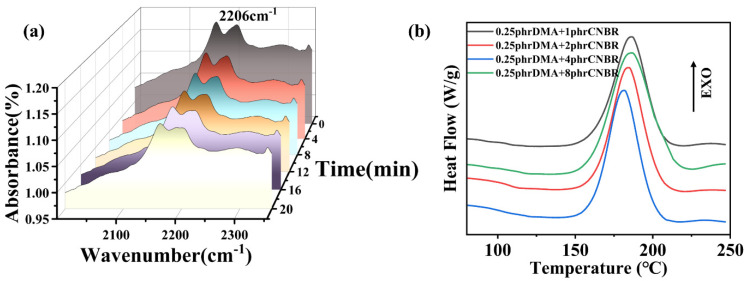
(**a**) The characteristic absorption peak of the nitrile group in CNBR changed at 160 °C. (**b**) DSC curves of 0.25phr DMA and different-ratio CNBR-modified epoxy coatings.

As we all know, the nitrile group in CNBR is a Lewis base, which theoretically acts as accelerator to enhance the curing reaction during the curing reaction process of epoxy coating. The DSC data also confirm that after the DMA is adsorbed by CNBR, the curing-promotion effect is better than that of pure tertiary amine catalysts. In order to investigated whether CNBR and DMA played a synergistic catalytic role in the process of the epoxy curing reaction, in situ diffuse reflection infrared spectroscopy (in-situ DRIFTs) was performed on the 1phr DMA-4CNBR/EP coating at a curing temperature of 160 °C. The absorbance of the nitrile group in CNBR is at 2206 cm^−1^ in Figure 6a; its absorbance intensity decreased rapidly from 1.17% to 1.13% in 4 min and then to 1.10% in 20 min, and the nitrile peak subsequently tended to the plateau from strong and narrow peaks. In order to further confirm the catalysis mechanism of the CNBR, 0.25phr DMA and different-ratio CNBR-modified epoxy coatings were prepared. Figure 6b showed DSC curves of 0.25phr DMA and different-ratio CNBR-modified epoxy coatings. As the proportion of rubber increased, the curing temperature of the coating decreased.

The in-situ DRIFTs results verified that rubber particles participated in the curing reaction of epoxy powder, which played the role of a curing accelerator, and showed that DMA and CNBR were synergistically catalyzed in the curing reaction. This DSC result could also indicate that rubber particles involved in the curing reaction, and rubber particles and DMA, played a synergistic catalytic role in the epoxy curing reaction. On the other hand, the decreased curing temperature may also be attributed to the good dispersion of rubber particles in epoxy coatings, and the dispersion of DMA in the matrix is improved by the loading of rubber particles on DMA, so the curing accelerator DMA had a better catalytic effect.

## 4. Conclusions

The rubber-composite nanoparticle-modified epoxy powder composite coating with low curing temperature and high toughness was successfully fabricated by surface adsorption and melt mixing. DMA-CNBR was uniformly dispersed in the epoxy coating with an average diameter 100 nm. Epoxy powder coatings loaded with 1phr DMA-4CNBR exhibited the lowest curing temperature and the highest mechanical performance compared to neat epoxy coatings. The DSC results showed that the curing temperature of 1phr DMA-4CNBR/EP could diminish by 38 °C compared to the neat epoxy-powder coating, while the activation energy of 1phr DMA-4CNBR/EP is 86.1 kJ/mol, nearly half of that of the neat epoxy coating (165.4 kJ/mol). The impact strength of 1phr DMA-4CNBR/EPs increased by 242%, while their adhesion strength increased by 108%. In addition, the Tg value of the 1phr DMA-4CNBR/EPs was increased.

## Figures and Tables

**Table 1:** Different formulations of the epoxy powder composite coatings.

**Ingredients (g)**	**DMA-4CNBR Content Relative to Epoxy Resin (wt%)**				
	0	0.25	0.5	0.75	1
Epoxy resin (604)	100	100	100	100	100
Curing agent (dicyandiamide)	6	6	6	6	6
Defoaming agent (benzoin)	0.3	0.3	0.3	0.3	0.3
Flatting agent (588)	0.7	0.7	0.7	0.7	0.7
DMA	0	0.25	0.5	0.75	1
CNBR	0	1	2	3	4

**Table 2:** Curing kinetics data of different content DMA-4CNBR/EP systems.

**Samples**	**DMA-4CNBR Content Relative to Epoxy Resin (wt%)**				
	0	0.25	0.5	0.75	1
**E_a_ (kJ/mol)**	165.4	105.5	99.1	88.2	86.1

## Data Availability

The data presented in this study are available on request from the corresponding author.
